# Climate change and public health in Germany – An introduction to the German status report on climate change and health 2023

**DOI:** 10.25646/11400

**Published:** 2023-06-01

**Authors:** Elke Hertig, Iris Hunger, Irena Kaspar-Ott, Andreas Matzarakis, Hildegard Niemann, Lea Schulte-Droesch, Maike Voss

**Affiliations:** 1 University of Augsburg Faculty of Medicine Augsburg, Germany; 2 Robert Koch Institute Centre for International Health Protection Berlin, Germany; 3 German Meteorological Service Research Centre Human Biometeorology Freiburg, Germany; 4 Robert Koch Institute Department of Epidemiology and Health Monitoring Berlin, Germany; 5 Federal Agency for Nature Conservation Division I 2.2 - Nature Conservation, Society and Social Issues Bonn, Germany; 6 Centre for Planetary Health Policy Berlin

**Keywords:** PUBLIC HEALTH, CLIMATE CHANGE, ADAPTATION, RESILIENCE, HEALTH

## Abstract

Global warming of 1.5°C and even 2°C is likely to be exceeded during the 21^st^ century. Climate change poses a worldwide threat and has direct and indirect effects on infectious diseases, on non-communicable diseases and on mental health. Not all people are equally able to protect themselves against the impacts of climate change; particularly populations that are vulnerable due to individual factors (children, older persons, those immunocompromised or with pre-existing conditions), social factors (the socially disadvantaged), or living and working conditions (e. g. people who work outdoors) are subject to an increased risk. Concepts such as One Health or Planetary Health provide a framework to frame both climate change itself and adaptation strategies or sets of actions for environmental human and animal health. Knowledge of climate change impacts has grown in recent years, and mitigation and adaptation strategies have been developed.

## 1. Preamble to the Status Report on Climate Change and Health 2023

Climate change poses one of the greatest threats to many people worldwide and has direct and indirect effects on communicable and non-communicable diseases. In view of the major health challenges, the German Federal Ministry of Health (BMG) is funding the project ‘Climate Change and Health – Status Report/Update with Advisory Board: Content, Communication, Working Methods’ (KlimGesundAkt), which is coordinated at the Robert Koch Institute (RKI, Germany’s national public health institute) and aims to update the 2010 Status Report on Climate Change and Health with a focus on Germany [1]. This update differs from the previous report in two ways:

(1) in the presence of an interdisciplinary and cross-institutional advisory board, which accompanies the entire planning and publication process of the updated status report,

(2) in the downstream target group-oriented communication and condensation of the results in the form of intuitive communication tools, including the formulation of recommendations for action.

The steps of this participatory and transparent process, which involved government authorities, university institutions and civil society, are outlined here.

The task of the interdisciplinary and cross-institutional advisory board was to deliberate on the report’s structure, scope and thematic focus. As a result, less space has been devoted to the causes of climate change than in the 2010 report, whereas other areas such as mental health and social inequality have been expanded or newly included. The coordination process took place in close cooperation with the internal RKI working group ‘Climate Change and Health’, which is independent of the project. This group brings together the RKI's knowledge of topics such as waterborne infections, heat-related mortality, vector-associated diseases, climate-related health behaviour, mental health surveillance and allergies, which is spread across various organisational units.

The status report will be published as a collection of articles in the Journal of Health Monitoring's Climate Change and Health series in three journal issues (Table 1). This reflects contemporary scientific publication practices and provides greater availability of the results to the public health community. Primary addressees are the interested professional public as well as decision-makers with a remit in public health. Since the Journal of Health Monitoring is published in German and English, and the English edition is archived at PubMed Central and accessible via PubMed search, international visibility is also ensured.

Authors with the desired expertise were identified on the basis of the topics to be addressed. A dual role for members of the advisory board as authors was encouraged. Additional scientists were consulted by the authors of the individual chapters where their expertise was needed. This process resulted in a group of more than 90 authors from over 30 research institutions and government agencies who are responsible for updating the Status Report on Climate Change and Health 2023.

Another important component of this project is targeted downstream science communication, which processes the results and recommendations contained in the individual articles through a wide range of tools (videos, fact sheets, social media content, digital channels, direct exchange formats) for specific target groups with public health relevance. This evidence-based communication strategy was developed at the RKI in close coordination with the advisory board and the Federal Centre for Health Education (BZgA). Some communication tools are to be tested and further developed in a participatory and iterative process involving target groups such as decision-makers and relevant stakeholders at the subnational level. Health communication is an important public health intervention in the field of climate change and health. Relevant decision-makers and the public need to know and assess increasing risks in order to act based on them.

## 2. Health, climate and climate change adaptation in Germany

The article presented here provides a general overview of climate change and health as the basis for the status report, particularly the climatic background and climate change-related health risks. Other articles that follow in this and two other Special Issues of the Journal of Health Monitoring (Table 1) summarise the current evidence in the various fields, briefly touched on here, in which climate change interacts with wildlife and the environment to affect human health.

In the long term, the health situation in Germany has been improving steadily. For example, since the early 1990s, life expectancy of people in Germany has increased by around four years to 83.4 years for women and by around six years to 78.5 years for men [2, 3]. Notwithstanding the increase in life expectancy, the effects of global climate change are increasingly becoming an important risk factor for health. Especially in the last few years, it has become apparent how fast climate change is reaching us. 2022 was the warmest year on record in Germany, and a pronounced spring drought occurred for the fourth year in a row [4]. Climate change will also make certain disasters more frequent, such as the heavy rain event that led to widespread flooding in July 2021, especially in Rhineland-Palatinate and North Rhine-Westphalia. Extreme events such as these can trigger disasters that are not the result of a single event, but must be understood as an interplay of different processes, modified by local conditions. These disasters may have an immediate impact, such as a physical effect on human health. However, cascading effects can also result in broader and far-reaching indirect health consequences, e. g. through lack of accessibility for emergency vehicles or through the development of chronic conditions or mental illness [5]. While climate change is most noticeable in Germany through a change in thermal stress, extreme weather events such as droughts, low water, heavy rain, storms, fires, and floods occur and can also have a strong impact on human health. In addition, there are indirect health effects moderated by natural systems, e. g. a pollen season prolonged by warmth with associated allergy burden, increased exposure to pollutants, and infections due to reduced hygiene after floods [6]. Infectious diseases not currently occurring in Germany to a significant extent are also expected to increase.

Climate change affects human health in part by altering ecosystems and, as one factor among many, exacerbates ongoing biodiversity loss. At the same time, ecosystems and their biodiversity play a role in the fluctuation of greenhouse gases and are an important lever of climate change adaptation [7]. The two crises must therefore be considered together in terms of their consequences for human health (Info box 1). There is a continuing need for research into the links between climate change, biodiversity and health.

So far, the Essential Public Health Functions (EPHFs) of the World Health Organization (WHO) [8] do not yet adequately reflect environmental changes or their role in climate change mitigation and adaptation. This article therefore draws on existing concepts of core public health functions [9] that link these functions to sustainability aspects and climate resilience. Concepts such as One Health and Planetary Health, as well as the public health core functions, are considered in relation to their utility for public health practice to enable health equity in climate change.

Even though climate change has a global dimension and ultimately, effective climate protection can only succeed globally, adaptation measures must be developed and implemented primarily at the regional or local level. Climate protection (mitigation) to prevent the progression of climate change is essential, but climate change adaptation is also important to enable people to remain healthy despite the changes. This status report focuses primarily on climate change adaptation in Germany.

## 3. Climatic changes

### 3.1 Climate development

When analysing long-term climate development in Germany, it becomes clear that climate change is already observable and perceptible. It is clearly due to the anthropogenic increase of greenhouse gases in the atmosphere (Info box 2). The most important anthropogenic greenhouse gas in this context is carbon dioxide (CO_2_), which caused a change in global radiative forcing of 2.16 W/m^2^ in the period from 1750 to 2019 (Info box 3). Together with other greenhouse gases, such as methane, nitrous oxide, and ozone, this results in total additional positive radiative forcing of 2.72 W/m^2^, which is associated with a global temperature increase of 1.2°C since the start of the 20^th^ century [25]. Since area-wide measurements began in 1881, the annual mean temperature in Germany has increased by 1.6°C degrees. The rate of temperature increase in Germany (as worldwide) has increased significantly over the past 50 years: since 1881, temperatures increased by an average of 0.12°C per decade; for the last 50 years, the warming rate has been more than three times as high, at 0.38°C per decade. Since the 1960s, each decade has been significantly warmer than the previous one. The rise in mean air temperatures is also likely to lead to more and more intense weather extremes in the coming years. The increase in heatwaves and dry spells has a strong impact on health [26].

**Info box 1 Importance of biodiversity for human health**

Biodiversity provides ecosystem services, direct and indirect contributions of ecosystems to human well-being, which are essential for human health. The dramatic loss of biodiversity thus poses a threat to human health.

A large number of scientific publications have identified correlations between biodiversity and human health. Research into causal relationships has been funded in Germany since August 2022 with a directive from the Federal Ministry of Education and Research [10].

The diversity of the plant spectrum provides us with medicine and food, regulates water and climate, and reduces environmental risks such as air pollution. In addition, there is a connection of biodiversity with allergies and immune diseases. For example, contact with a microbe-rich environment in childhood, such as through agriculture, has been identified as a protective factor against allergies [11, 12].

Biodiversity also plays a role in infectious diseases. For example, the risk of pandemics may increase due to human destruction and alteration of ecosystems [13]. Unsustainable consumption, agricultural intensification, and wildlife trade lead to increased contact between humans and wildlife, triggering a variety of zoonotic diseases. Thus, biodiversity conservation has an important preventive role.

**Contribution of biodiversity to mental health and human well-being**

Spending time in nature has a positive effect on human well-being and mental health; this has been well researched for some time. Urban green spaces, gardens, forests, and bodies of water provide opportunities for recreation [14], stress reduction [15], and social interactions.

However, it has not yet been clearly established whether this effect is also stronger the more diverse the nature surrounding humans is, since socioeconomic factors, cultural background and aesthetic preference of the study participants, but also the definition of biodiversity and health of the respective study play a role [16–18].

Cultural ecosystem services also make an important contribution to mental health [19]. People value diverse ecosystems and some species for their beauty, feel connected to them, and identify with a particular environment.

**Synergies between climate change adaptation, public health strategies, and conservation**

**Figure 1:**
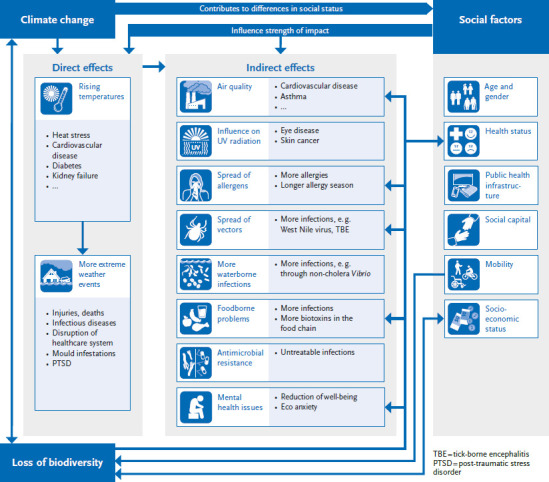
Direct and indirect effects of climate change on health Illustration: Robert Koch Institute

Given the complex interrelationships between climate change, biodiversity loss, and human health (Figure 1), there are important synergies between climate change adaptation, public health measures, and nature conservation [20]. Nature-based solutions can achieve positive outcomes in terms of both health and adaptation to climate change, so-called co-benefits [21]. One example is the expansion of urban green space and urban blue infrastructure. This includes roadside trees and street greenery, greening of facades and green roofs, and larger green spaces (parks, playgrounds) that promote recreation, air pollution control, and microclimate [22]. To simultaneously serve biodiversity conservation, this greenery should be as diverse as possible. Socioeconomically disadvantaged people in particular benefit from nature-based health interventions in cities [23]. In view of increasing urbanisation worldwide – it is predicted that 68% of the world's population will already live in cities by 2050 [24] – the development of urban, diverse greenery should therefore not be underestimated as an important public health measure.

According to the German coordination office of the Intergovernmental Panel on Climate Change (IPCC), global air temperatures will continue to rise until at least mid-century under all emissions scenarios considered. Global warming of 1.5°C and even 2°C is likely to be exceeded during the 21^st^ century unless drastic reductions in CO_2_ and other greenhouse gas emissions occur in the coming years [27]. Many changes in the climate system are amplified in direct relation to increasing global warming [25]. Natural factors and internal variability will modulate human-induced forcing, especially at regional scales and in the near future. It is important to consider these modulations when planning for the full range of potential impacts. As global warming continues, projections indicate that simultaneous and multiple modifications of climatic impact drivers (CIDs) will increasingly occur in nearly all regions. Regional climatic impacts are predominantly negative in nature, although there are some regions that could benefit from climate change. Changes in several CIDs would be more widespread at 2°C compared to 1.5°C global warming, and even more widespread and/or pronounced at higher levels of warming. Effects with low probability of occurrence – such as ice sheet collapse, abrupt changes in ocean circulation, some compound extreme events, and warming substantially beyond the range assessed as very likely – cannot be ruled out and are part of the risk assessment [25].

From a natural science perspective, limiting human-induced global warming to a certain level requires limiting cumulative CO_2_ emissions, achieving at least net zero CO_2_ emissions, along with strong reductions in other greenhouse gas emissions. Strong, rapid, and sustained reductions in greenhouse gas emissions would also limit the warming effect resulting from declining air pollution, since aerosols (especially particulate matter) have predominantly negative radiative forcing and their projected decline by mid-century will have an additional warming effect [28].

**Info box 2 Greenhouse effect**

The greenhouse effect is a natural process that significantly determines the temperature on Earth. Water vapour and carbon dioxide are the two main natural greenhouse gases that cause the global mean temperature to be a comfortable 15°C, rather than -19°C, as it would be without the presence of the Earth's atmosphere. Increasing the concentrations of atmospheric trace gases such as carbon dioxide, methane, and nitrous oxide creates an additional (anthropogenic) greenhouse effect. This results in further warming of the lower atmosphere and affects the entire climate system.

**Info box 3 Radiative forcing**

The additional radiative forcing is the change in net insolation (incoming solar radiation minus outgoing radiation, expressed in watts per square meter, W/m^2^) at the top of the troposphere, the lowest layer of the Earth's atmosphere. This change occurs due to anthropogenic climate change, primarily by increasing the concentration of greenhouse gases in the atmosphere. The additional radiative forcing is defined as the change from the year 1750. The numbers in the representative concentration pathways (RCP) climate scenario names, e. g. RCP2.6 or RCP8.5, refer to the expected change in radiative forcing of 2.6 W/m^2^ and 8.5 W/m^2^, respectively..

### 3.2 Climate models and climate projections

Climate models provide information about the future development of the climate. The calculated future projections depend, among other factors, on the assumed development of human society. In order to be able to represent these potential global developments, scenarios have been developed over the past decades from which different development paths of emission and concentration scenarios of greenhouse gases and aerosols can be derived. The oldest scenario family (special report on emissions scenarios, SRES) reflects the state of knowledge at the turn of the millennium, the subsequent generation of scenarios (representative concentration pathways, RCP) was developed for the IPCC’s Fifth Assessment Report, and now considers other factors such as climate change mitigation and adaptation [29]. The latest generation is called shared socioeconomic pathways (SSP) and focuses on changing socioeconomic factors, such as population, economic growth, education, urbanisation, and the pace of technological development [30]. In doing so, the SSP identify five different ways in which the world could develop without climate policies and how different levels of climate action could be achieved. In doing so, the climate mitigation targets of the RCP are combined with the SSP. The RCP set pathways for greenhouse gas concentrations and thus the amount of warming that could occur by the end of the century. The SSP, on the other hand, provide the framework within which emissions reductions are achieved (or not achieved) [31].

The five socioeconomic development paths of the SSP scenarios (SSP1 to SSP5), are associated with additional radiative forcing (1.9 to 8.5 W/m^2^). Scenarios with low or very low greenhouse gas emissions (SSP1-1.9 and SSP1-2.6) lead to detectable positive impacts on greenhouse gas concentrations as well as air quality in a matter of years compared to scenarios with high and very high greenhouse gas emissions (SSP3-7.0 or SSP5-8.5). When comparing these contrasting scenarios, discernible differences between global air temperature trends begin to emerge from natural variability within about 20 years.

## 4. Impact of climate change on health

The Climate Impact and Risk Assessment 2021 for Germany lists eight climate risks in the field of human health, which are also in line with the structure of this status report and the following sections [32]: heat stress, UV-related health damage, allergic reactions, potentially harmful micro-organisms and algae, distribution and change in abundance of possible vectors, respiratory issues due to air pollution, injuries and deaths as a result of extreme events, and effects on the healthcare system.

These impacts of climate change on humans and the environment are highly dependent on geographic region, human use of the environment, and social determinants [33]. Any person can be affected by diseases that are influenced by, at different temporal scales, weather and climate; nevertheless, there are parts of the population that are much more vulnerable to the health consequences (like heatwaves) of climate change and, in some cases, respond more strongly. In particular, these are individuals vulnerable due to their age or those weakened by immune or other pre-existing conditions. In addition, there are groups of people who are exposed to health-threatening situations longer and more frequently than others due to occupational or private activities [33]. Interdependencies between age, gender, work/housing conditions and location, income or poverty as well as education status also play a role here [34]. In a coming article of this status report on climate change and health, this topic is discussed in more detail (Bolte et al. [35]).

Accordingly, different groups have very different needs and requirements of the healthcare system. This shows that the climate impacts described above not only affect individuals, but also have a significant impact on the healthcare system and its players, and thus have organisational and economic components. Health in particular has many cross-connections with other fields of action [36].

Health is not only an individual concern, but above all a social task. Various prevention and health promotion measures can enable people to behave in a way that promotes health by adapting to climate change and its effects. These include interventions such as educational campaigns, like the website ‘Klima-Mensch-Gesundheit’ (Climate-Human-Health) of the BZgA. However, these measures should always be combined with a change in people’s living conditions, because health behaviour is difficult to enforce against resistance from the social environment or living conditions. These so-called structural preventive measures include, for example, the expansion of infrastructure and services offered (e. g. bicycle paths, climate-friendly cafeteria food, shaded public areas, access to urban greenery) or pricing policies (e. g. promoting climate-neutral construction). These complex interventions require the involvement of many social groups and sectors beyond health, for example by cooperating with actors from the transport, construction or environmental sectors. Such an approach requires that health is considered in all policies, so that health-promoting lifestyles actually become the easiest for people in everyday life, as the WHO demands (‘Make the healthy way the easy way’) [37].

Many consequences of global warming interact with each other and can reinforce each other through feedback effects. In addition to the direct effects of climate change on human health – in Western Europe primarily through increased occurrence of extreme weather events such as heatwaves – indirect effects can also be observed, which are brought about through changes in natural systems (atmosphere, bio-, hydro-, cryo- and pedosphere) (Figure 1). Weather conditions determine the local meteorological conditions to a large extent, as well as air and radiation hygiene. For example, long-lasting summer high-pressure weather conditions not only lead to thermal stress due to high air temperatures, but also to increased exposure to ozone and UV radiation. Climate change causes changes to infectious diseases whose pathogens are transmitted via blood-sucking arthropods, infections that are water or food-related, and changes in the area of allergenic plants and animals. In addition, progressive climate change can cause further changes relevant to health, like an increase in drought episodes, which indirectly affect health, e. g. via insufficient water supply, crop failures or the increase in forest fires, or an increase in flooding events, which can lead to the spread of certain pathogens. Both are already a major challenge today [38–40].

The various impacts of climate change on health are briefly touched on here and addressed in detail in subsequent articles in this and the next Special Issue in the Climate Change and Health series of articles, with reference to both communicable diseases and non-communicable diseases, including mental health impairments.

### 4.1 Impact of climate change on communicable diseases

#### Vector and rodent associated infectious diseases

According to a study by McIntyre et al. [41], nearly two-thirds of the human and domestic animal disease pathogens found in Europe are climate sensitive. Climatic conditions favour, among other things, the outbreak of vector-associated diseases such as chikungunya, dengue, and West Nile fever in Europe and contribute to the further geographic spread of vectors that transmit the causative agents of Lyme borreliosis and tick-borne encephalitis.

Transmission of vector-associated pathogens requires an introduced or established vector population, a pathogen, and appropriate environmental and climatic conditions throughout the pathogen transmission cycle. Environmental and climatic conditions affect each of these areas, from vector survival and abundance to pathogen growth and survival in vector organisms, to vector activity and sting frequencies, to human exposure to disease vectors.

Even if, for example, the introduction of *Aedes albopictus* (Asian tiger mosquito) is primarily favoured by globalisation, especially along transport routes [42], climate change is associated with the active potential spread of vectors and pathogens, which is why a further shift of certain tick species to higher latitudes and altitudes and a further geographical spread of mosquito and sandfly species should be expected in Germany in the coming years [43]. Another article in this status report is dedicated in detail to vector- and rodent-associated diseases in climate change (Beermann et al. [44])

#### Waterborne infections and intoxications

Waterborne pathogens may also be subject to the influence of climate change. The increase in sea surface temperatures, as evidenced by measurements in the North Sea and Baltic Sea (in the North Sea, temperature increased by about 1.3°C over the last 50 years) [45], will continue in the future and accelerates the proliferation of the bacterial genus *Vibrio*, for example [46, 47]. *Vibrio* infections mainly manifest as wound infections and diarrhoeal diseases. Climate warming with accompanying increased water temperatures could lead to higher *Vibrio* concentrations, making an increase in infections more likely [47–49]. Projections indicate that the sea surface temperature of the North Sea will warm by 1°C to 3°C by the end of the 21^st^ century, and that of the Baltic Sea by 3°C to 4°C, with strongest warming rates in the northern part of the Baltic Sea [50]. In addition, extreme precipitation events may lead to outbreaks of waterborne diseases [49]. The topic of waterborne infections and intoxications is dealt with in more detail in another article in this status report (Dupke et al. [51]).

#### Foodborne infections and intoxications

Foodborne infections and intoxications also play a role in the context of climate change, as the incidence of associated diseases can be affected by temperature changes. Examples include bacterial gastrointestinal infections caused by the mostly foodborne pathogens *Campylobacter* and *Salmonella*. Transmission to humans usually occurs through food. *Salmonella* infections increase linearly with air temperature by 5 to 10% per °C [52]. Thus, longer summers allow increased transmission of foodborne pathogens [49]. More detailed findings can be found in another paper in this status report (Dietrich et al. [53]).

#### Antimicrobial resistance (AMR)

One link between health and climate change that has received little attention is antibiotic-resistant infections [54]. Bacteria that cause infections in humans can develop resistance to antibiotics. Resistance against antimicrobial agents (in bacteria and other microbes) causes significant morbidity and mortality worldwide, posing enormous challenges to health systems and basic public health functions globally. Antibiotic resistance in bacteria is thought to develop mainly under the selection pressure of antibiotic use. However, other factors, such as climate change, may also contribute to the increase in antibiotic resistance. MacFadden et al. [55] reported that a 10°C increase in temperature in experimental laboratory settings was associated with an increase in antibiotic resistance in the common pathogens *Escherichia coli* (+4.2%), *Klebsiella pneumoniae* (+2.2%), and *Staphylococcus aureus* (+2.7%). In another contribution to this status report, Meinen et al. [56] provide a systematic review on AMR in climate change.

### 4.2 Impact of climate change on non-communicable diseases and mental health

#### Health impact due to air pollutants

In recent decades, air quality in Germany has improved considerably thanks to targeted air pollution control measures [57]. However, if emissions remain constant, there would be an increase in ground-level ozone and particulate matter concentrations as a result of climate change. Warmer summers and, in particular, an increase in extreme temperature events favour the formation of ground-level ozone, as stagnant air circulation during pronounced high-pressure weather conditions can cause ozone to accumulate and allow peak levels to occur over several days [58, 59]. Increased particulate matter exposure, e. g. due to increasingly dry soils and more frequent vegetation fires, can cause cardiovascular disease in addition to impaired lung function and serious lung diseases such as asthma and lung cancer. Likewise, there is a significant relationship between cardiovascular mortality and levels of ground-level ozone, with even short-term exposure to ozone increasing health risk and moderately high levels of ozone being associated with increasing rates of myocardial infarction [60, 61]. Further health impacts arise from increased heat stress, especially in combination with increased air pollutants [62, 63]. In a contribution to this status report, Breitner-Busch et al. [64] provide an overview of climate change-related health effects from air pollutants that are particularly relevant for Germany, and explain the effects of air pollutants in conjunction with air temperature. Furthermore, an overview of limit, target and guideline values in the current context of the air situation in Germany is given, and the current WHO guideline values are discussed. Corresponding recommendations for the public health sector are presented.

#### Health impact due to heat

Heat events usually occur over large areas and affect individual groups, especially younger and older people, but are also cross-sectoral [65]. Heatwaves will increase in terms of intensity, duration, and frequency (Figure 2). The characteristics of heatwaves, their number, duration, and intensity, as well as their temporal occurrence within a year, are relevant for estimating the health burden during and after high thermal stress. In particular, the duration of a heatwave can increase mortality, and the disproportionate increase in long heatwaves can therefore lead to a sharp increase in the number of deaths [66]. For example, RCP scenarios for Germany project an increase in mortality from chronic lower respiratory diseases (CLRD) during long heatwaves (≥10 days) of up to 150% by mid-century. Depending on the scenario, there could be an increase of between 260% (RCP4.5) to a maximum of 540% (RCP8.5) by the end of the century. According to RCP4.5 and RCP8.5, mortality from ischemic heart disease (IHD), which involves narrowing of the coronary arteries, could be 90% (RCP4.5) or 150% (RCP8.5) higher between 2021 and 2050, and as much as 330% (RCP4.5) higher to a maximum of 900% (RCP8.5) between 2068 and 2097 [67]. This indicates that even under moderate climate change, significant health effects from heat can be expected. In a subsequent article of this status report Winklmayr et al. [68] address the health effects of high temperatures, which particularly affect older persons and people with certain pre-existing conditions.

#### Health impacts from extreme weather events

Increases in intense rainfall can cause devastating floods that directly affect the lives and health of the population and health infrastructure. The intensity of rainfall in Germany and Western Europe has already increased by up to 19% due to climate change. Flooding events, such as those in western Germany and Belgium in 2021, have become up to nine times more likely. If global warming approaches the 2°C threshold, this will be directly reflected in precipitation intensities (increase of up to 6%) and flood probabilities [70].

However, increased dryness and drought must also be expected, especially in the summer months. Modelling has shown that with a warming of 3°C by the end of the century, twice as many days of drought are to be expected in Germany [71]. Historical data show that various regions of Germany are already suffering from increasing drought [72]. In addition to stressful situations in agriculture, low water levels and falling groundwater levels, this can also have an impact on air quality. Dry soils contribute to a worsening of air quality by dust and particulate matter due to drifting. Prolonged drought also increases the time pollen stays airborne, and more frequent forest fires contribute locally to increased exposure to particulate matter. Drought stress in plants also reduces the uptake of ozone, thereby increasing the ground-level ozone concentration that is harmful to health, and thus increasing the incidence of respiratory diseases [73]. In another contribution to this status report, Butsch et al. [74] provide an overview of health impacts due to increased extreme weather events occurring under climate change. According to this article, such indirect or long-term meteorological consequences can be countered by risk management and disaster relief in order to mitigate the health consequences of extreme events as far as possible, especially for vulnerable groups.

#### Health effects due to increased allergen exposure

Climate change has an influence on allergen exposure. For example, increased pollen production and an earlier pollen season support the occurrence and increase the frequency of pollen-associated allergic respiratory diseases [75]. In addition, higher temperatures and an increase in the air’s CO_2_ content can lead to an increase in the allergenicity of pollen and thus cause stronger allergic reactions [76]. Climate change-induced changes in vegetation zones also allow alien species to colonise and spread in areas where they were not previously native. For Europe, for example, the ragweed plant with its high allergenic potential is a cause for concern [6, 75, 77]. Bergmann et al. [78], in another contribution to this status report, deal in detail with the topic of climate change-induced changes in allergen exposure and their health consequences, show the connection to other exposures such as air temperature and air pollutants, and give recommendations for action.

#### Health effects due to altered UV radiation exposure

Climate change has an influence on ground-level UV radiation and the annual UV radiation dose. For Germany, the effects of greenhouse gases on stratospheric ozone and especially cloud cover play a decisive role here. Projections for stratospheric ozone and cloud density are subject to very large uncertainties. However, in recent years, a significant increase in sunshine duration has been recorded in Germany and, consequently, an increase in the daily sums of erythemally effective UV radiation annual dose [79, 80]. The occurrence of UV radiation-related diseases of the skin and eyes, including cancers, depends not only on the prevailing ground-level UV irradiance in the environment (ambient UV radiation) of people and the UV radiation annual dose, but is also decisively determined by the exposure behaviour of people. The current state of knowledge on effects of climate change on UV radiation exposure and health consequences are described in a contribution to this status report by Baldermann et al. [81].

#### Impact of climate change on mental health

In addition to increasing general concern among many people about the future of the planet, climate change may have other consequences for mental health. The effects of climate change-related weather events and natural disasters on mental health have been known for some time. They cause problems such as sleep disorders, stress, anxiety, depression, and the development of post-traumatic stress disorder and suicidal ideation [82]. However, less research has been done on the psychological and emotional consequences generated by awareness of the slow and gradual changes in the environment caused by human-induced climate change and what measures can effectively protect vulnerable groups in particular. Studies show that people experience feelings of loss, helplessness, and frustration due to the threat of climate change – a condition now referred to as eco anxiety [83]. In a further contribution to this status report, Gebhardt et al. [84] address this issue and examine the topics of extreme weather events, temperature increase, perception and inner-psychological processing of climate change, psychological-sociological aspects, as well as the ability to act and resilience factors.

## 5. Conceptual frameworks for addressing the climate crisis

The concepts of One Health and Planetary Health have gained momentum since the early 2000s; they can provide a framework for addressing the climate crisis. Inherent in their systemic-holistic approach is a broader view of possible solutions to problems of environmental change and health.

Both concepts are subject to constant further development and thus do not represent rigid monoliths. As a result, both approaches can be instrumental in achieving many of the goals of the United Nations’ ‘2030 Agenda for Sustainable Development’ (Sustainable Development Goals), thus significantly strengthening that agenda and ultimately improving environmental health [85]. The impacts of climate change affect variously interrelated systems, such as the relationship between the economy, energy, the environment, and health, and thus can only be understood and resolved across these fields [27]. In order to improve the protection of human health, the interrelationships between human and animal health and healthy ecosystems must therefore be considered more seriously in a wide range of policy areas.

### 5.1 One Health

The concept of One Health was proposed by Schwabe [86] in 1964, when he coined the term ‘One Medicine’ for the areas encompassing human and veterinary health. Since then, the concept of One Health has been extended to include the environmental aspect: humans are a part of the animal kingdom, which in turn is embedded in a common environment [87]. The ‘Berlin Principles’ recently further enriched the One Health concept [88]. They emphasise the institutional strengthening needed to ensure the translation of science-based knowledge into policy and practice, as well as the need for action in addressing the climate crisis.

The One Health High-Level Expert Panel (OHHLEP) was convened by WHO and other organisations in order to initiate the implementation of the One Health approach from theory to practice. Four areas were deemed essential: communication, coordination, collaboration, and capacity building [89]. The balance between sectors and disciplines should be considered, sociopolitical and multicultural parity, socioecological balance, human responsibility, as well as transdisciplinarity and multisectoral collaboration across all relevant disciplines.

Through the One Health concept, a holistic view of all affected areas can reinforce synergistic approaches along responsible administrative and executive levels, in order to achieve solutions that can reduce the effects of climate change or implement adaptation strategies. For example, the costs resulting from the emergence of new zoonoses could be significantly lower if they were identified early on as potential zoonoses in animals rather than appearing later in humans [90].

### 5.2 Planetary Health

Planetary Health is a concept that relates human health to political, economic, and social systems, as well as the ecological boundaries of our planet [91]. It highlights the dominance and impact of human activities on shaping our environment, and calls for the associated responsibility and recognition of planetary boundaries. By transforming toward health within planetary boundaries, ecological stress limits are no longer exceeded, while current and future generations are enabled to live healthy, dignified lives in safety through effective and sustainable political, social, and economic systems [92]. Planetary Health is a health narrative based on sustainability and the critique of growth economies, which builds on interdisciplinary as well as intersectoral engagement with the complex relationships between and within ecosystems. The understanding of Planetary Health goes beyond the isolated consideration of environment and climate. In Germany, political Planetary Health recommendations for the field of climate change and health were formulated in the 2021 Lancet Countdown Policy Brief as follows [93]:

(1) the systematic and widespread implementation of heat-health action plans to reduce heat-related health risks,

(2) the reduction of the CO_2_ footprint of the German healthcare sector and

(3) the integration of climate change and health/Planetary Health in education and training of health professionals.

### 5.3 Climate change mitigation and adaptation as tasks for the health care system

Evidence and knowledge about the impact of climate change have continued to grow in recent years, and climate change mitigation and adaptation strategies have been developed both globally and nationally.

Pivotal at the global level is the Paris Agreement, which was adopted at the 21^st^ Conference of the Parties of the United Nations Framework Convention on Climate Change (COP21) in December 2015. The signatory states pledged to limit global warming to well below 2°C, but preferably to 1.5°C, compared with pre-industrial levels. At COP26 in Glasgow, all states agreed to accelerate the global energy transition away from coal combustion; at COP27 2022 in Egypt, this acceleration was not evident.

In 2022, the German G7 presidency placed climate change and health on the political agenda of those seven industrialised countries that have joined together as the ‘Group of 7’, thus promoting national and international attention to climate-neutral and climate-resilient health systems.

At the federal level, the Climate Change Act guides the actions of the healthcare system in enforcing climate protection. This law was amended by a decision of the Federal Constitutional Court. The decision stated that requiring only a mild reduction in CO_2_ consumption of the current generation and allowing them to use up most of the remaining CO_2_ budget is unconstitutional as it leaves future generations with a high reduction burden, and exposes them to extensive losses of freedom [94]. The German government has set up a climate protection programme and is preparing a climate change adaptation strategy [95]. A national prevention plan is also in preparation, which, among other things, will introduce concrete measures against climate and environment-related health damage. The National Prevention Conference outlines tasks facing individual actors in this context [96]. A climate adaptation act will create a framework for implementing the national climate change adaptation strategy with measurable targets in cooperation with the federal states.

In 2008, the German government set the strategic framework with the German Strategy for Adaptation to Climate Change (Deutsche Klimaanpassungsstrategie, DAS), to counter the effects of climate change with a focus on 16 fields of action [97]. A network of all federal ministries and 28 higher federal authorities is involved. Measurable targets are being developed in many clustered topics, including health.

Most political activities related to climate change adaptation involve protection against heat. According to a resolution of the 93^rd^ conference of German federal health ministers, heat-health action plans are to be drawn up in federal states and municipalities nationwide by 2025 [98]. The recommendations, which can be used as a model, were developed jointly by the Federal Ministry for the Environment, Nature Conservation, Nuclear Safety and Consumer Protection, the BMG and the federal states [99].

Even in the absence of concrete targets and measures on how the healthcare system can become climate-neutral and climate-resilient, some transformative action is evident at the level of healthcare actors and institutions. For example, the 125^th^ German Medical Assembly called for a national strategy for climate-friendly healthcare [100]. In December 2022, the BMG, leading organisations in the health care sector, the federal states and municipal umbrella organisations signed the Climate Pact for Health and declared their intention to work together for adaptation and mitigation in the healthcare sector [101]. It is important to note that mandates in the German healthcare system are very heterogeneous, and responsibility is distributed in a complex manner among different levels and within the self-governance of involved actors.

Health must not be considered alone in the development and implementation of mitigation and adaptation measures; health must be considered in all departments in the sense of ‘Health in all Policies’. Co-benefits are achieved through measures that are both good for the climate and the environment, and promise public health benefits through risk reduction or health promotion. In particular, the areas of renewable energy (reducing emissions and improving air quality), active rather than motorised mobility (reducing emissions, improving air quality, and increasing physical activity), and plant-based diets (reducing emissions and reducing non-communicable diseases) hold great potential for these co-benefits [102]. Because of the synergies between climate change adaptation, the improvement of human health, and biodiversity conservation, more partnerships should be formed between urban planning, landscape design, conservation, and health and other sectors.

### 5.4 Transformation toward a resilient public health system

It is apparent that a systemic view via the One Health or Planetary Health approaches is necessary for climate change adaptation and climate protection.

Brown and Westaway [103] describe how successfully dealing with adversity and challenges can entail a reorganisation or transformation of systems in which adaptive functions are optimised. The transformative consequence of resilient behaviour is also reflected in current thinking about environmental change and socioecological systems.

In particular, the WHO definition of resilience provides a comprehensive description in this context. The WHO defines resilience as the ability of a health system to prepare for, cope with, and learn from shock events, among other things, while maintaining the core functions of the health system [104]. Ideally, resilience is not a return to the original state, but an evolution to a better state [105]. Transformation as part of health system resilience can refer to the system’s ability to change practices, re-design certain services or public health programs to be more accessible, or it can refer to medical and technological breakthroughs. Resilience at the system level can be strengthened by introducing new financing mechanisms; this can increase the economic sustainability of the system and its ability to anticipate and counter potential future crises.

In addition to enabling health systems to better cope with climate change-related challenges, health care institutions must also do their part to mitigate climate change. The healthcare sector contributes significantly to the emission of greenhouse gases worldwide. The healthcare sector’s share of German greenhouse gas emissions ranges from 5.2% [106] to 6.7% [107], depending on the estimate. Currently, there is no legal obligation for standardised reporting of greenhouse gas emissions in the German healthcare sector. Only a few healthcare facilities document their emissions voluntarily [93].

Relevant areas for reducing the ecological footprint of hospitals, the pharmaceutical industry and other healthcare facilities include the manufacture and supply chains of drugs and medical devices, construction, energy supply, nutrition or communal catering, waste reduction and separation, climate-friendly alternatives in consumables (from anaesthetic gases to single-use instruments to office materials), and environmentally friendly workplace health promotion and occupational safety. Physicians enjoy a high degree of trust among the population and therefore have an important societal role to play in raising awareness and changing behaviour in favour of co-benefits, e. g. diets that emit little CO_2_ and also promote health [108].

To address and adapt to the challenges of environmental determinants of health, an integrated and evidence-based approach is needed in healthcare, public health, and across sectors, informed by good governance, appropriate management mechanisms, high-level political will, and adequate human, technical, technological, and financial resources. The WHO is currently further developing approaches that incorporate climate change into health concepts, such as the Essential Public Health Functions (EPHFs), a model for assessing and developing public health structures and their functions [9, 109]. These core functions for public health provide guidance to public health systems; however, publications that apply to the European region have so far given too little consideration to environmental and climate aspects. The German Zukunftsforum Public Health (future forum on public health) published a ‘Call for and to Action: Climate Change and Public Health’ in the summer of 2022, which formulated recommendations for public health stakeholders and policy makers on this topic. The core functions of public health are addressed, but not yet fully formulated [110].

Although the public health core functions for Germany and Europe have not yet been formulated to include climate change and environmental aspects, the core functions of the Pan American Health Organization (PAHO) of the WHO already refer to climate resilience and sustainability [9]:

(1) Monitoring and evaluation of health and well-being, equity, and social determinants of health to determine their impact on environmental public health

(2) Environmental health surveillance of environmental hazards, exposures, health risks, and risk management measures

(3) Promotion and management of environmental health research and knowledge

(4) Development and implementation of environmental health policies and promotion of legislation in this area

(5) Participation and social mobilisation to promote communication and action on environmental determinants of health

(6) Development of human resources for environmental public health

(7) Use and management of essential medicines and health technologies in an environmentally safe and sustainable manner to protect public health

(8) Efficient and equitable financing of environmental public health

(9) Equitable access to health care facilities that are climate resilient and environmentally sustainable

(10) Equitable access to environmental public health interventions that promote health, reduce risk factors, and promote healthy behaviours

(11) Including the environmental public health dimension in the management and promotion of interventions on social determinants of health.

The complete elaboration of a concept on public health and environmental and climate factors is still pending. However, the individual articles and, in particular, the final article of this Status Report on Climate Change and Health can make a substantial contribution to this and use the preliminary work described. The following articles list recommendations for action, the implementation of which can reduce the impact of climate change for the public health sector. To conclude the series of articles, these recommendations will be revisited in a separate article and related to the public health core functions mentioned above in order to provide guidance to public health actors in strengthening the resilience of the healthcare system.

The authors of all the articles published in this series thus present an up-to-date, actionable report focused on Germany that stresses our ability to act in the face of the threats posed by climate change and provides actors with a solid basis for concrete action. The scientific evidence is overwhelming, the status report provides orientation – action must now be taken.

## Key statement

- Global man-made climate change will continue to progress and affect human health.
- Biodiversity contributes directly and indirectly to human health.
- Protecting and restoring biodiversity is an important public health measure.
- The direct and indirect effects of climate change on human health are highly dependent on region, social factors, and human behaviour.
- Climate change has an impact on people’s physical and mental health. Not all people can protect themselves equally well against climate change impacts.
- Basic public health functions will be severely challenged by climate change impacts.
- A climate-resilient health system could be guided by internationally established Essential Public Health Functions.

## Figures and Tables

**Figure:**
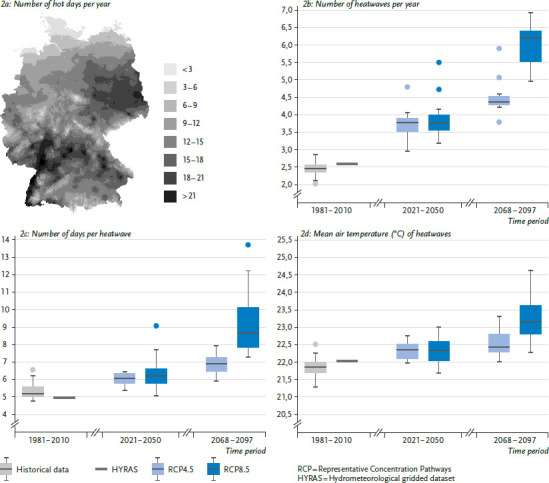
Figure 2a Number of hot days per year. Regional distribution of the number of hot days with a maximum temperature of at least 30°C, 2011–2020 Source: German Meteorological Service Figure 2b Number of heatwaves per year Figure 2c Number of days per heatwave Figure 2d Mean air temperature (°C) of heatwaves 2b – 2d in Germany, based on historical (measured) datasets, HYRAS (gridded dataset) [69], and projections under the RCP4.5 and RCP8.5 climate scenarios Source: Own representation based on Schlegel et al. [67]

**Table 1:** Overview of all articles in three Special Issues in the Journal of Health Monitoring's Climate Change and Health series

Issue	Topic
**Introduction to the topic of climate change and health**	
1	Introduction to the Status Report on Climate Change and Health
**Impact of climate change on infectious disease…**	
1	…by vector- and rodent-associated diseases.
1	…due to waterborne infections and intoxications
1	…due to foodborne infections and intoxications
1	…through antimicrobial resistance
**Impact of climate change on non-communicable disease…**	
2	…due to temperature changes
2	…due to extreme weather events
2	…due to UV radiation
2	…due to allergen exposure
2	…due to air pollutants
**Impact of climate change on mental health.**	
2	Impact of climate change on mental health.
**Cross-cutting issues related to climate change and health**	
3	Social determinants of the health impacts of climate change
3	Target group-specific communication
3	Options for action and implications
